# Effects of Radioactive ^56^MnO_2_ Particle Inhalation on Mouse Lungs: A Comparison between C57BL and BALB/c

**DOI:** 10.3390/ijms242417605

**Published:** 2023-12-18

**Authors:** Zhaslan Abishev, Bakhyt Ruslanova, Saulesh Apbassova, Dariya Shabdarbayeva, Nailya Chaizhunussova, Altai Dyusupov, Almas Azhimkhanov, Kassym Zhumadilov, Valeriy Stepanenko, Sergey Ivanov, Peter Shegay, Andrey Kaprin, Masaharu Hoshi, Nariaki Fujimoto

**Affiliations:** 1 Department of Pathological Anatomy and Forensic Medicine, Semey Medical University, Semey 071400, Kazakhstan; zhaslan_love@mail.ru (Z.A.); baharuslanova@gmail.com (B.R.); saulesh.apbasova@smu.edu.kz (S.A.); dariya_kz67@mail.ru (D.S.); 2 Department of Public Health, Semey Medical University, Semey 071400, Kazakhstan; n.nailya@mail.ru; 3 Rector’s Office, Semey Medical University, Semey 071400, Kazakhstan; altay.dyusupov@smu.edu.kz; 4 National Nuclear Center of the Republic of Kazakhstan, Kurchatov 071100, Kazakhstan; azim-hanov@nnc.kz; 5 Department of Nuclear Physics, L.N. Gumilyov Eurasian National University, Astana 010000, Kazakhstan; zhumadilovk@gmail.com; 6 A. Tsyb Medical Radiological Research Centre—Branch of the National Medical Research Radiological Centre of the Ministry of Health of the Russian Federation, 249031 Obninsk, Russia; valerifs@yahoo.com (V.S.); oncourolog@gmail.com (S.I.); 7 National Medical Research Radiological Centre of the Ministry of Health of the Russian Federation, 249036 Obninsk, Russia; dr.shegai@mail.ru (P.S.); mnioi.nauka@mail.ru (A.K.); 8 The Center for Peace, Hiroshima University, Hiroshima 730-0053, Japan; mhoshi@hiroshima-u.ac.jp; 9 Research Institute for Radiation Biology and Medicine, Hiroshima University, Hiroshima 734-0037, Japan

**Keywords:** environmental residual radiation, radiation-induced lung injury, internal radiation exposure, mouse strain differences

## Abstract

The effects of residual radiation from atomic bombs have been considered to be minimal because of its low levels of external radioactivity. However, studies involving atomic bomb survivors exposed to only residual radiation in Hiroshima and Nagasaki have indicated possible adverse health effects. Thus, we investigated the biological effects of radioactive dust of manganese dioxide 56 (^56^MnO_2_), a major radioisotope formed in soil by neutron beams from a bomb. Previously, we investigated C57BL mice exposed to ^56^MnO_2_ and found pulmonary gene expression changes despite low radiation doses. In this study, we examined the effects in a radiation-sensitive strain of mice, BALB/c, and compared them with those in C57BL mice. The animals were exposed to ^56^MnO_2_ particles at two radioactivity levels and examined 3 and 65 days after exposure. The mRNA expression of pulmonary pathophysiology markers, including Aqp1, Aqp5, and Smad7, and radiation-sensitive genes, including Bax, Phlda3, and Faim3, was determined in the lungs. The radiation doses absorbed in the lungs ranged from 110 to 380 mGy; no significant difference was observed between the two strains. No exposure-related pathological changes were observed in the lungs of any group. However, the mRNA expression of Aqp1 was significantly elevated in C57BL mice but not in BALB/c mice 65 days after exposure, whereas no changes were observed in external γ-rays (2 Gy) in either strain. In contrast, Faim3, a radiation-dependently downregulated gene, was reduced by ^56^MnO_2_ exposure in BALB/c mice but not in C57BL mice. These data demonstrate that inhalation exposure to ^56^MnO_2_ affected the expression of pulmonary genes at doses <380 mGy, which is comparable to 2 Gy of external γ-irradiation, whereas the responses differed between the two mouse strains.

## 1. Introduction

To understand the health effects of atomic bombs in Hiroshima and Nagasaki in 1945, the contribution of residual radiation has been considered to be unimportant because of its low radioactivity as an external radiation source [1]. Residual radiation has two sources: radioactive fallout containing fission products from bombs and radioisotopes generated by neutron beams on the ground. Although the former enters the atmosphere and “falls out” elsewhere, the effects of the latter have also become a concern because a report has suggested that individuals who moved to these cities soon after the detonation and were exposed to only residual radiation from the ground suffered from acute radiation syndromes [2]. More recently, Otani et al. reported that there were significant increases in solid cancer mortality risks in early entrants after the Hiroshima atomic bombing, suggesting the importance of residual radiation created by the neutron beam [3].

Manganese 56 (^56^Mn) is a primary radioisotope produced in the soil after an atomic bomb explosion [4]. We thus investigated the biological effects of ^56^MnO_2_ particles on laboratory rats (Wistar) and mice (C57BL) [5,6]. The effects of these particles on the lung, a primary target organ of inhalation, were examined. The absorbed pulmonary radiation doses were approximately 100 mGy in rats and 250 mGy in mice. Although these animals were exposed to ^56^MnO_2_ particles with similar radiation activity, organ doses in mice were higher than those in rats because β-rays from nearby organs contribute more due to the smaller body size in mice [7]. No pathological alterations related to ^56^MnO_2_ particle exposure were observed in either species. However, the expression of pulmonary genes, including aquaporins (AQPs), which are pathophysiological markers of lung function, was altered by ^56^MnO_2_ exposure, suggesting that internal exposure to residual radioactive particles has significant biological effects [5,6].

Radiation sensitivity differs among different mouse strains. The BALB/c strain is known to be “radiation-sensitive” compared with the C57BL strain based on the lethality of acute doses of whole-body irradiation [8]. This difference could be explained by the radiation sensitivity of the intestine, where the stem cells of the intestinal epithelia more rapidly recover from irradiation in C57BL [9]. Similarly, the radiation sensitivity in the hematopoietic system was also higher in BALB/c mice. However, when radiation-induced fibrosis is considered, C57BL mice are fibrosis-prone, whereas BALB/c mice are resistant [10,11]. Comparing the effects of ^56^MnO_2_ exposure between different strains is useful in elucidating the mechanism of radiation toxicity. In this study, male C57BL and BALB/c mice were exposed to ^56^MnO_2_ particles activated by neutrons. The effects on the lungs were investigated; moreover, basic biological parameters, including organ weights, were examined. The mRNA expression of AQPs, which are markers for lung lesions such as pulmonary edema [12,13], was measured. The global gene expression analysis demonstrated that the expression of genes involving cell cycle and apoptosis, such as Bax, Phil3, and Faim3, is valuable for biodosimetry [14,15,16]. We determined the expression of these genes in the lungs to confirm the radiation responses.

## 2. Results

### 2.1. Estimated Absorbed Doses of Internal Irradiation in the Lungs

The accumulated doses in the organs of mice exposed to ^56^MnO_2_ were previously estimated [7]. The absorbed doses in the lungs were 140 ± 20 and 340 ± 70 mGy for the Mn56 × 1 and Mn56 × 3 groups, respectively, in C57BL mice, whereas they were 110 ± 30 and 380 ± 70 mGy, respectively, in BALB/c mice.

### 2.2. Body and Organ Weights

The initial average body weights were 29.2 ± 0.3 g in C57BL mice and 27.2 ± 0.4 g in BALB/c mice. No significant difference in the average body weights was observed among the groups. The final body weights and relative weights of the thymus, spleen, lung, heart, liver, kidney, and testis 3 and 65 days after exposure are summarized in Table 1. On day 3, the thymus weights decreased in both the Mn56 × 3 and Co60 groups in BALB/c mice; however, they were reduced in the Co60 group only in C57BL mice. On day 65, the testis weights increased in both the Mn56 × 3 and Co60 groups in C57BL mice only.

### 2.3. Histology of the Lung

**Figure 1:**
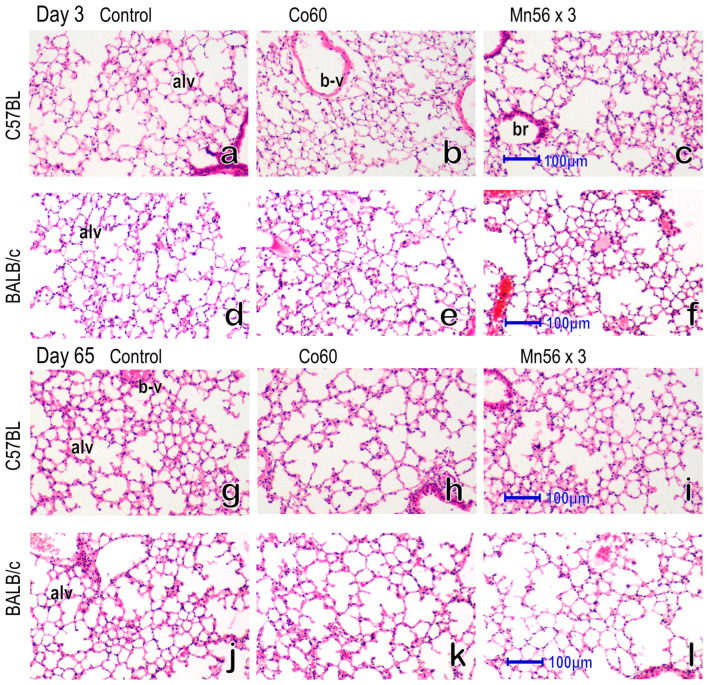
The mouse lungs on day 3 (**a**–**f**) and day 65 (**g**–**l**) after radiation exposure, HE staining. (**a**–**c**) and (**g**–**i**) indicate C57BL mice, and (**d**–**f**) and (**j**–**l**) indicate BALB/c mice. No significant histological alternations were noted in the lungs among the Control (**a**,**d**,**g**,**j**), Co60 (**b**,**e**,**h**,**k**), and Mn56 × 3 groups (**c**,**f**,**i**,**j**) in either mouse strain. The normal structure of the alveoli (alv), bronchioles (br) and blood vessels (b-v) were observed. Original magnification × 400. Bars indicate 100 µm.

The representative histology of the lung via hematoxylin and eosin (HE) staining 3 and 65 days after exposure in the control, Co60, and Mn56 × 3 groups of both strains is shown in Figure 1. No notable changes in the lungs were observed among the groups in either strain. There were no signs of alveolar wall thickening.

### 2.4. Expression of Biodosimetry Marker Genes

**Figure 2:**
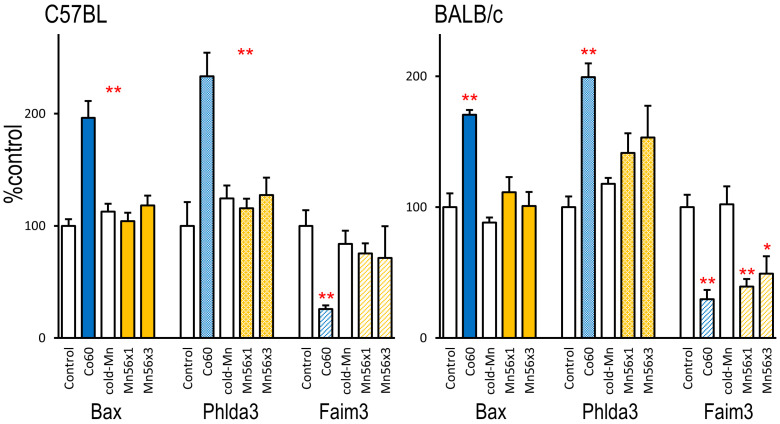
Relative mRNA expression levels of Bax, Phlda3, and Faim3 in the lungs of C57BL (**left**) and BALB/c mice (**right**) 3 days after exposure to ^56^MnO_2_ particles (Mn56 × 1 and Mn56 × 3), nonradioactive MnO_2_ particles (Cold-Mn), or 2 Gy of external γ-rays (Co60). * *p* < 0.05 or ** *p* < 0.01 vs. Control or Cold-Mn. The expression of Bax and Phlda3 increased in the Co60 group in both strains, whereas the mRNA levels of Faim3 decreased. ^56^MnO_2_ exposure also strongly suppressed Faim3 expression but only in BALB/c mice.

The pulmonary mRNA levels of three radiation-sensitive genes, Bax, Phida3, and Faim3, are summarized in Figure 2. Three days after exposure, radiation-responsive increases in Bax and Phlda3 were evident in the Co60 group in both C57BL and BALB/c mice, whereas no changes were observed in the Mn56 groups. The expression of Faim3 decreased following Co60 irradiation in both strains. Faim3 levels were also reduced in the Mn56 × 3 group but only in BALB/c mice. Sixty-five days after exposure, the expression of these biodosimetry maker genes returned to the control levels.

### 2.5. Gene Expression of Aqp1, Aqp5, and Smad7

**Figure 3:**
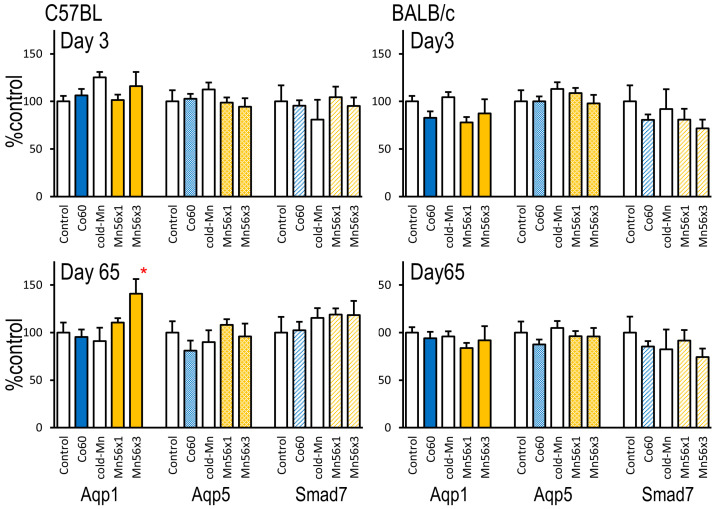
Relative mRNA expression levels of Aqp1, Aqp5, and Smad7 in the lungs 3 and 65 days after exposure to ^56^MnO_2_ particles (Mn56 × 1 and Mn56 × 3), nonradioactive MnO_2_ particles (Cold-Mn), or 2 Gy of external γ-rays (Co60) in C57BL (**left**) and BALB/c (**right**) mice. * *p* < 0.05 vs. Control or Cold-Mn.

Changes in Aqp1, Aqp5, and Smad7 mRNA levels after exposure are summarized in Figure 3. In C57BL mice, the expression levels of Aqp1 were similar among the groups on day 3; however, they became significantly higher in the Mn56 groups on day 65. In BALB/c mice, no changes in the expression of Aqp1 were observed at either timepoint. No changes in the gene expression of Aqp5 or Smad7 were observed in either mouse strain.

## 3. Discussion

Regarding the health effects of atomic bombs in Hiroshima and Nagasaki in 1945, there have been concerns about the effects of the residual radiation produced by the bombs on soil [2]. A recent cohort study demonstrated that solid cancer mortality risks were significantly higher in early entrants after the Hiroshima atomic bombing without exposure to any direct radiation, suggesting that residual radiation contributes to the health effects of the bombs [3]. Possible radionuclides activated by atomic bombs’ neutron beams are ^24^Na, ^28^Al, ^31^Si, ^32^P, and ^56^Mn [4]. Because ^56^Mn is a significant residual radiation source produced by atomic bombs, we investigated the biological effects of ^56^MnO_2_ on laboratory rats and mice [5,6]. Our studies suggested that exposure to ^56^MnO_2_ particles induced more prominent physiological responses than external γ-irradiation in both rats and mice, although mice were less sensitive to internal exposure than rats [5]. The radiation sensitivity greatly differs among different mouse strains [8]. Understanding these differential responses helps to evaluate the health effects of ^56^MnO_2_ exposure [17]. Therefore, in this study, we focused on investigating the differential responses to ^56^MnO_2_ exposure in two mouse strains: C57BL and BALB/c.

BALB/c mice are known to be “radiation-sensitive” because of their lower 50% lethal dose (LD50) caused by whole-body irradiation [8]. The strain difference in acute lethality could be attributed to the radiation sensitivity of the intestine, where the intestinal epithelial stem cells are more susceptible to radiation in BALB/c mice [9]. A reduction in thymus weight is a well-known indicator of radiation exposure [18]. In this study, the thymus weights were significantly reduced in both the Mn56 × 3 and Co60 groups in BALB/c mice; however, the thymus weights were reduced only in the Co60 group in C57BL mice, proving the higher radiation sensitivity of the BALB/c strain. This also indicates that ^56^MnO_2_ exposure induces significant biological responses in BALB/c mice. The thymus weight losses recovered afterward, as previously reported [19].

Gene expression changes involving cell cycles and apoptosis regulation, such as Ccng1 and Bax, are useful for radiation dosimetry [14,15]. After irradiation, these genes’ expression is regulated through p53 [20,21]. A recent study of a more comprehensive gene expression analysis in radiation-exposed C57BL mice identified highly sensitive marker genes, including Phlda3 and Faim3 [16]. In this study, we used these gene expression markers to examine the biological effects of ^56^MnO_2_ exposure in the lungs. In the Co60 group, 2 Gy of external irradiation increased the expression levels of Bax and Phlda3 and suppressed the expression of Faim3 3 days after exposure; however, these changes disappeared on day 65 in both mouse strains. In the Mn56 × 3 and Mn56 × 1 groups, the radiation-dependent suppression of Faim3 expression was evident in BALB/c mice but not in C57BL mice, indicating strain-dependent responses.

High doses of thoracic irradiation lead to radiation pneumonitis and lung fibrosis in animals and humans [22,23,24]. Because lung injuries are the main dose-limiting factors in radiotherapy, they have been intensively investigated to determine the underlying mechanisms [25]. TGF-β and the Smad signaling pathway regulate wound healing and subsequent fibrosis [26,27,28]. Studies have found that irradiation could disrupt this process by altering the expression of Smad genes, resulting in lung injuries [29]. Our previous rat study demonstrated that exposure to ^56^MnO_2_ significantly increased Smad7 mRNA levels [6]. However, in mice, the mRNA levels of Smad7 were unaffected by ^56^MnO_2_ or γ-ray exposure in either strain.

The mRNA expression of AQPs, which are water-selective channel proteins, is also a valuable marker related to lung injuries [12]. Aqp1 is expressed in subepithelial connective tissues and capillaries, whereas Aqp5 is located in the lung epithelium [30]. These proteins are essential in maintaining lung structure and function by controlling the water permeability of the plasma membrane. In radiation- or chemical-induced lung injuries, changes in the mRNA expression of AQPs represent pathophysiological alterations [13]. The gene expression of Aqp1 increased in pulmonary edema models, whereas the expression of Aqp1 and Aqp5 decreased in acute lung injury models [31,32,33]. In our previous study involving C57BL mice, ^56^MnO_2_ exposure resulted in a late increase in the expression of Aqp1, indicating pathophysiological alterations without obvious histological changes [5]. This study confirmed the late effects of ^56^MnO_2_ exposure on Aqp1 expression as Aqp1 increased 65 days after exposure in the Mn56 × 3 group but not in the Co60 group. The effects of ^56^MnO_2_ on Aqp1 expression were not observed in BALB/c mice. Although BALB/c mice are generally considered “radiation-sensitive,” this sensitivity differs depending on the biological outcomes. When radiation-induced pulmonary fibrosis is concerned, C57BL mice are fibrosis-prone, whereas BALB/c mice are resistant to fibrosis development [12,13]. Increases in the gene expression of Aqp1 on day 65 may be related to the fibrosis-prone nature of C57BL mice, which could be the key biological effect of ^56^MnO_2_ exposure. Other studies of lung injury in murine models also have suggested the crucial role of Aqp1. In chemically or physically induced lung injury models of mice, Aqp1 expression is increased [34,35], although it reduces in the early stage of injury [36,37]. The longer-term effects of ^56^MnO_2_ should be explored in the future.

The behaviors of specific gene expression changes may be involved in strain-dependent lung damage. A cDNA microarray analysis of different strains of mice locally irradiated in the thorax demonstrated that two genes, growth differentiation factor 15 and hyaluronan synthase 1, were highly expressed in C57BL but not in C3H or A/J mice [38]. In cigarette smoke models of lung injury in mice, pulmonary transcriptomes after exposure were found to differ between C57BL and AKR strains [39]. Another study examining the effect of ozone on the lung found that mouse strain-dependent responses to the exposure were associated with airway macrophage transcriptional activity [40].

Mn is neurotoxic at high doses [41]. It can lead to lung inflammatory reactions [42,43]. This study found no signs of pulmonary inflammation in the Cold-Mn group, suggesting that the exposed MnO_2_ level was nontoxic. The pulmonary level of MnO_2_ calculated based on the inhaled radioactivity of ^56^Mn was <26 µg/g lung tissue in this study.

Based on the changes in the gene expression of Faim3 in BALB/c mice and Aqp1 in C57B mice, internal exposure to ^56^Mn appeared to have stronger biological effects on the lung tissue, considering that the pulmonary doses were <380 mGy in the ^56^Mn groups versus the external dose of 2 Gy in the Co60 group. Our study also revealed strain differences in radiation sensitivity to internal exposure to ^56^MnO_2_ particles. However, it is not clear which stain is more relevant to understanding the human health effect. The stronger biological effect of ^56^Mn may be explained by the peculiarities of the spatial dose distributions in the tissue from beta particle radiation from inhaled ^56^Mn microparticles [44]. Further studies are needed to determine whether the radioactive microparticle effect contributes to the stochastic effects, such as increasing cancer risks.

## 4. Materials and Methods

### 4.1. Animals

Specific pathogen-free male C57BL mice (7 weeks old) and BALB/c mice (7 weeks old) were purchased from the Kazakh Scientific Center of Quarantine and Zoonotic Diseases, Almaty, Kazakhstan. The animals were housed individually in plastic cages (one mouse/cage) and maintained with free access to the basal diet and tap water. Animals were maintained in a conventional type of facility (room temperature: 19–22 °C, relative humidity: 30–70%, a 12-h light–dark cycle). Mice of each strain were randomly divided into five groups at 10 weeks of age: Mn56 × 1 (*n* = 14), Mn56 × 3 (*n* = 14), Cold-Mn (*n* = 11), Co60 (*n* = 11), and Control (*n* = 11). The Mn56 × 1 and Mn56 × 3 groups were exposed to two activities of ^56^MnO_2_ particles (100 mg): 2.4 × 10^8^ and 8 × 10^8^ Bq, respectively. The Co60 group received 2 Gy of external ^60^Co γ-ray whole-body irradiation. All animal groups were brought to the ^56^Mn exposure facility to maintain the same environment. Five mice/group were necropsied on post-exposure day 3, and 6 mice/group were necropsied on day 65. The mice were euthanized by removing whole blood from an abdominal aorta under anesthesia with isoflurane (Fujifilm Wako Pure Chemical Co., Tokyo, Japan). The thymus, spleen, lung, heart, liver, kidney, and testis were dissected and weighed. Pieces of the lungs were stored in RNA Save solution (Biological Industries Ltd., Beit Alfa, Israel) for RNA extraction, and part of the lungs was fixed in 10% formalin. Paraffin sections of 4-µm thickness were prepared and stained with HE. The animal study was approved by the Animal Experiment Ethics Committee of Semey Medical University, Semey, Kazakhstan (document #3-30.11.2018). The study was conducted in compliance with the Animal Research: Reporting of In Vivo Experiments guidelines.

### 4.2. Irradiation and Dosimetry

^56^Mn is a radioisotope that decays into ^56^Fe, emitting both β- and γ-rays with a radiation half-life of 2.58 h. Details of irradiation using ^56^MnO_2_ particles and internal dose estimation have been previously described [7]. Briefly, MnO_2_ particles (Rare Metallic Co., Tokyo, Japan; chemical purity is 99.99%; particle diameters range from 1 to 19 µm) were radioactivated by a neutron beam in the Baikal-1 nuclear reactor at the National Nuclear Center, Kurchatov, Kazakhstan. The ^56^MnO_2_ particles were then air-pressure-sprayed into a sealed exposure box containing 14 mice. After 1 h of exposure, three mice per group were euthanized for dosimetry. The γ-rays of dissected organs were measured. Absorbed fractions of energy from the β- and γ-irradiation of ^56^Mn in organs were calculated using the Monte Carlo code (version MCNP-4C) and a mathematical mouse phantom. For external whole-body γ-ray irradiation, the Teragam K-2 irradiator (UJP Praha, Praha-Zbraslav, Czech Republic) was used (2.0 Gy at 1.0 Gy/min).

### 4.3. Measurement of mRNA Levels Using Quantitative Reverse Transcription Polymerase Chain Reaction (qRT-PCR)

qRT-PCR was performed as previously described [6]. The measured mRNA levels were normalized with reference to the β-actin mRNA levels. Specific primer sets for genes are listed in Table 2.

### 4.4. Statistical Analysis

All values are expressed as means ± standard error of the mean. Dunnett’s test was used to compare the values between the Mn56 and Cold-Mn groups or between the Co60 and Control groups. The R package “SimComp” was used (http://cran.r-project.org, accessed on 8 November 2023). The original data and the statistical analysis results are listed in Tables S1–S3.

## 5. Conclusions

The effects of internal exposure to ^56^MnO_2_ particles on the lungs were examined in two mouse strains: C57BL and BALB/c. Inhalation exposure to ^56^MnO_2_ resulted in absorbed doses of 110–380 mGy in the lungs. It increased the expression of Aqp1 in C57BL mice 65 days after exposure, whereas Faim3 expression decreased in BALB/c mice on day 3, indicating strain differences in radiation responses. These effects were comparable to or higher than those of external γ-irradiation of 2 Gy, suggesting that exposure to ^56^MnO_2_ particles has significantly greater biological effects than external irradiation on the lungs. This may indicate the possible roles of residual radiation in the health effects of atomic bomb exposure.

## Figures and Tables

**Table 1:** Body weights and relative organ weights in mice exposed to ^56^MnO_2_ (Mn56 × 1 and Mn56 × 3), nonradioactive MnO_2_ (Cold-Mn), and external γ-rays.

	Body Weights (g)	Thymus (g/kg bw)	Spleen (g/kg bw)	Lung (g/kg bw)	Heart (g/kg bw)	Liver (g/kg bw)	Kidney (g/kg bw)	Testis (g/kg bw)
Day 3/C57BL								
Control	28.7 ± 1.0	1.7 ± 0.27	2.9 ± 0.30	10.2 ± 1.1	5.7 ± 0.12	52.5 ± 3.7	15.4 ± 0.58	6.8 ± 0.28
Co60	28.8 ± 1.2	0.8 ± 0.09 *	2.8 ± 0.32	8.5 ± 0.73	5.6 ± 0.37	60.5 ± 3.5	15.6 ± 0.60	7.1 ± 0.64
Cold-Mn	29.0 ± 0.9	1.4 ± 0.18	3.5 ± 0.30	9.4 ± 0.36	5.8 ± 0.23	51.9 ± 1.0	15.2 ± 0.33	6.5 ± 0.33
Mn56x1	29.5 ± 1.4	1.3 ± 0.08	4.7 ± 0.21 *	9.0 ± 0.38	5.9 ± 0.18	55.0 ± 2.3	14.8 ± 0.50	5.9 ± 0.73
Mn56x3	29.4 ± 1.2	1.4 ± 0.10	3.3 ± 0.38	9.6 ± 0.55	4.8 ± 0.13 *	55.2 ± 1.5	14.8 ± 0.82	6.5 ± 0.82
Day 3/Balb/c								
Control	26.3 ± 1.6	1.4 ± 0.14	3.8 ± 0.38	9.2 ± 0.70	5.6 ± 0.20	49.3 ± 2.5	17.0 ± 0.70	8.8 ± 0.61
Co60	26.2 ± 1.9	0.9 ± 0.09 *	3.1 ± 0.40	8.8 ± 0.51	5.6 ± 0.24	56.1 ± 1.7	18.2 ± 0.52	9.2 ± 0.63
Cold-Mn	27.5 ± 1.8	1.2 ± 0.11	4.8 ± 0.32	8.7 ± 0.54	5.5 ± 0.24	46.9 ± 2.7	17.8 ± 0.58	9.1 ± 0.58
Mn56x1	27.0 ± 2.1	1.4 ± 0.14	4.1 ± 0.23	8.8 ± 0.50	5.9 ± 0.21	52.2 ± 1.6	16.2 ± 0.67	8.5 ± 0.87
Mn56x3	27.3 ± 1.9	1.0 ± 0.06 *	4.5 ± 0.53	9.0 ± 0.50	5.4 ± 0.12	56.9 ± 2.1	17.4 ± 0.50	7.5 ± 0.87
Day 65/C57BL								
Control	36.0 ± 1.3	1.6 ± 0.09	2.9 ± 0.28	7.4 ± 0.67	5.2 ± 0.25	51.1 ± 2.53	15.2 ± 0.59	5.3 ± 0.23
Co60	32.6 ± 0.6	1.3 ± 0.10	3.4 ± 0.33	8.3 ± 0.45	6.0 ± 0.36	48.2 ± 2.44	16.0 ± 0.63	6.6 ± 0.22 *
Cold-Mn	33.6 ± 1.0	1.4 ± 0.11	3.0 ± 0.29	7.2 ± 0.52	5.8 ± 0.25	49.2 ± 2.42	16.4 ± 1.06	6.2 ± 0.59
Mn56x1	35.4 ± 2.1	1.3 ± 0.14	3.2 ± 0.40	9.4 ± 0.77	5.5 ± 0.21	47.6 ± 2.5	14.7 ± 0.80	5.9 ± 0.95
Mn56x3	33.9 ± 1.5	1.6 ± 0.14	2.8 ± 0.31	8.3 ± 0.38	5.7 ± 0.33	43.7 ± 1.74	15.5 ± 0.46	6.9 ± 0.61 *
Day 65/Balb/c								
Control	29.8 ± 2.5	1.2 ± 0.17	3.6 ± 0.22	8.9 ± 0.85	6.8 ± 1.02	49 ± 2.5	18.0 ± 0.57	8.7 ± 0.68
Co60	32.1 ± 2.3	1.2 ± 0.11	3.1 ± 0.28	7.3 ± 0.42	5.6 ± 0.20	45.9 ± 3.6	17.0 ± 0.94	7.0 ± 0.68
Cold-Mn	28.6 ± 1.7	1.1 ± 0.09	3.2 ± 0.33	8.4 ± 0.47	5.6 ± 0.24	44.9 ± 2.1	17.8 ± 0.49	9.1 ± 0.36
Mn56x1	28.4 ± 1.6	1.2 ± 0.09	3.9 ± 0.54	9.7 ± 0.59	5.9 ± 0.31	49.2 ± 3.3	18.4 ± 0.88	7.1 ± 1.07
Mn56x3	27.5 ± 1.6	1.2 ± 0.1	4.0 ± 0.33	9.1 ± 0.8	6.6 ± 0.74	48 ± 1.4	19.1 ± 0.74	9.1 ± 0.99

* *p* < 0.05 vs. Control.

**Table 2:** Quantitative PCR primers.

Gene	GenBank Accession #	Q-PCR Primer Sequences (5′ -> 3′)	
		Forward	Reverse
Bax	NM_007527	CGTGGACACGGACTCCCCCC	TGATCAGCTCGGGCACTTTA
Phlda3	NM_013750	AAGCCGTGGAGTGCGTAGAG	GTCTGGATGGCCTGTTGATTC
Faim3	NM_026976	TTCATGAGCAAAGGACACGC	CACCAGTCCCAAAAGAACCAG
Aqp1	NM_007472	ACCTGCTGGCGATTGACTACA	CATAGATGAGCACTGCCAGGG
Aqp5	NM_009701	CTCCCCAGCCTTATCCATTG	CACGATCGGTCCTACCCAGA
Smad7	AF015260	TTGCTGTGAATCTTACGGGAAG	GGTTTGAGAAAATCCATTGGGT
Actb	NM_007393.5	CTGTCCCTGTATGCCTCTGGTC	TGAGGTAGTCCGTCAGGTCCC

## Data Availability

Data is contained within the article and supplementary materials.

## Supplementary Materials

The supporting information can be downloaded at: https://www.mdpi.com/article/10.3390/ijms242417605/s1.
