# Supplementary material for: Effects of Radioactive 56MnO2 Particle Inhalation on Mouse Lungs: A Comparison between C57BL and BALB/c
Source: Int J Mol Sci. 2023 Dec 18;24(24):17605. doi: 10.3390/ijms242417605 (PMC10743477; doi:10.3390/ijms242417605)
Supplement: Supplementary file 1 [file ijms-24-17605-s001.zip › TableS3.pdf]

C57BL-Lung

			AQ1			AQ5			Smad7		
Day 3		Average		x10/bact			x10/bact			x10/bact	
Mn56x1-3I	C1-01	26.0	2.57	0.99		1.61	0.62		1.33	0.51	
	1-02	21.4	1.85	0.86		1.11	0.52		0.84	0.39	
	1-03	21.9	2.54	1.16		1.52	0.69		1.03	0.47	
	1-04	13.0	1.44	1.11		0.84	0.65		0.93	0.72	
	1-05	15.2	1.41	0.93	1.01	1.09	0.72	0.64	0.98	0.65	0.55
					0.05			0.03			0.06
Mn56x3-3I	2-01	16.1	1.95	1.22		1.06	0.66		0.75	0.47	
	2-02	18.8	1.27	0.67		0.97	0.51		0.66	0.35	
	2-03	26.8	3.07	1.15		2.01	0.75		1.56	0.58	
	2-04	20.0	3.20	1.60		1.39	0.69		1.25	0.62	
	2-05	10.6	1.22	1.15	1.16	0.47	0.44	0.61	0.51	0.48	0.50
					0.15			0.06			0.05
Co60-3D	3-01	27.8	2.90	1.04		2.05	0.74		1.39	0.50	
	3-02	20.6	1.77	0.86		1.20	0.58		0.91	0.44	
	3-03	15.2	1.84	1.21		1.09	0.72		0.85	0.56	
	3-04	11.8	1.16	0.98		0.83	0.70		0.69	0.58	
	3-05	15.1	1.81	1.20	1.06	0.88	0.59	0.66	0.65	0.43	0.50
					0.07			0.03			0.03
coldMn-3I	4-01	20.9	3.05	1.46		1.86	0.89		1.29	0.62	
	4-02	17.4	2.06	1.19		1.31	0.75		0.80	0.46	
	4-03	23.5	2.83	1.21		1.62	0.69		1.31	0.56	
	4-04	30.0	3.74	1.25		1.82	0.61			0.00	
	4-05	9.8	1.12	1.14	1.25	0.69	0.71	0.73	0.48	0.49	0.43
					0.05			0.05			0.11
C-3D	5-01	5.3	0.47	0.88		0.40	0.75		0.18	0.33	
	5-02	14.0	1.54	1.10		0.82	0.58		0.59	0.42	
	5-03	13.7	1.56	1.14		0.74	0.54		0.78	0.57	
	5-04	11.3	1.13	1.00		0.53	0.47		0.52	0.46	
	5-05	8.7	0.74	0.85	1.00	0.77	0.88	0.65	0.74	0.84	0.53
				1.09	0.06		0.66	0.08		0.50	0.09
Day 65											
Mn56x1-6	C1-06	47.6	2.74	0.57		3.64	0.76		1.88	0.40	
	1-07	41.3	2.47	0.60		2.75	0.67		1.38	0.33	
	1-08	45.4	3.05	0.67		2.60	0.57		1.81	0.40	
	1-09	50.0	2.88	0.58		3.40	0.68		1.76	0.35	
	1-10	31.8	1.75	0.55		1.67	0.53		0.88	0.28	
	1-11	41.6	2.96	0.71	0.61	2.93	0.70	0.65	1.42	0.34	0.35
					0.03			0.04			0.02
Mn56x3-6	2-06	40.6	3.92	0.97		3.65	0.90		1.86	0.46	
	2-07	27.5	3.05	1.11		1.89	0.69		1.24	0.45	
	2-08	30.1	2.18	0.72		1.74	0.58		1.00	0.33	
	2-09	39.1	2.25	0.57		2.02	0.52		0.84	0.22	
	2-10	32.3	1.99	0.62		1.56	0.48		0.76	0.23	
	2-11	16.9	1.19	0.70	0.78	0.51	0.30	0.58	0.67	0.40	0.35
					0.09			0.08			0.04
Co60-65D	3-06	41.4	3.40	0.82		2.59	0.63		1.75	0.42	
	3-07	35.0	2.06	0.59		1.78	0.51		0.91	0.26	
	3-08	28.3	1.51	0.53		1.29	0.46		0.69	0.24	
	3-09	20.2	0.61	0.30		1.65	0.82		0.68	0.34	
	3-10	39.6	1.89	0.48		2.31	0.58		1.57	0.40	
	3-10	29.8	0.93	0.31	0.51	0.78	0.26	0.54	1.13	0.38	0.34
					0.08			0.08			0.03
coldMn-65	4-06	41.6	2.72	0.65		3.19	0.77		1.27	0.31	
	4-07	16.2	0.75	0.46		0.82	0.50		0.54	0.33	
	4-08	15.9	0.89	0.56		0.81	0.51		0.46	0.29	
	4-09	23.4	1.51	0.65		1.07	0.46		0.56	0.24	
	4-10	11.4	0.50	0.44		0.42	0.37		0.27	0.23	

BalbC-Lung

		bact	AQ1			AQ5			Smad7		
Day 3		Average		x10/bact			x10/bact			x10/bact	
Mn56x1-3D	B1-01	28.5	4.00	1.40		6.00	2.10		1.75	0.61	
	1-02	16.1	2.55	1.58		3.61	2.24		0.92	0.57	
	1-03	14.5	2.22	1.53		2.37	1.64		0.68	0.47	
	1-04	18.5	3.09	1.67		4.38	2.37		1.35	0.73	
	1-05	13.7	1.93	1.41	1.52	3.22	2.36	2.14	0.80	0.59	0.59
					0.05			0.13			0.04
Mn56x3-3D	2-01	16.1	2.91	1.80		3.66	2.27		1.06	0.66	
	2-02	19.6	3.06	1.56		3.58	1.83		0.83	0.42	
	2-03	13.2	2.43	1.84		2.52	1.91		0.78	0.59	
	2-04	15.8	3.14	1.99		3.66	2.32		0.94	0.60	
	2-05	18.9	2.51	1.33	1.71	2.50	1.32	1.93	0.70	0.37	0.53
					0.12			0.18			0.06
Co60-3D	3-01	32.9	4.66	1.42		7.34	2.23		1.59	0.48	
	3-02	26.8	3.66	1.37		5.00	1.87		1.44	0.54	
	3-03	19.7	3.58	1.82		3.33	1.69		1.32	0.67	
	3-04	17.2	3.27	1.90		3.93	2.29		1.18	0.69	
	3-05	13.0	2.03	1.57	1.61	2.31	1.78	1.97	0.75	0.58	0.59
					0.11			0.12			0.04
coldMn-3D	4-01	18.4	3.87	2.10		4.29	2.32		1.29	0.70	
	4-02	31.3	6.16	1.97		7.87	2.52		2.10	0.67	
	4-03	18.8	4.34	2.30		4.02	2.13		1.59	0.84	
	4-04	21.3	3.98	1.87		4.64	2.18		1.20	0.56	
	4-05	13.0	2.51	1.94	2.04	2.56	1.97	2.23	0.77	0.60	0.68
					0.08			0.09			0.05
C-3D	5-01	25.9	5.05	1.95		7.51	2.91		2.03	0.79	
	5-02	23.8	4.88	2.05		4.29	1.80		1.70	0.71	
	5-03	17.4	2.63	1.51		3.68	2.11		1.39	0.80	
	5-04	10.6	2.89	2.73		2.00	1.89		0.87	0.83	
	5-05	7.4	1.11	1.50	1.95	0.84	1.14	1.97	0.41	0.55	0.73
					0.23			0.28			0.05
Day 65				x10/bact			x10/bact			x10/bact	
Mn56x1-65D	B1-06	41.2	5.06	1.23		8.81	2.14		1.82	0.44	
	1-07	58.3	4.69	0.80		8.90	1.53		2.11	0.36	
	1-08	26.9	2.94	1.09		4.90	1.82		1.07	0.40	
	1-09	26.7	3.02	1.13		4.16	1.56		1.34	0.50	
	1-10	32.0	4.90	1.53		6.78	2.12		1.95	0.61	
	1-11	12.8	1.66	1.29	1.18	2.21	1.72	1.81	0.77	0.60	0.49
					0.10			0.11			0.04
Mn56x3-65D	2-06	28.4	4.76	1.67		5.60	1.97		1.27	0.45	
	2-07	35.7	5.04	1.41		6.28	1.76		1.84	0.52	
	2-08	33.2	4.09	1.23		6.58	1.98		1.23	0.37	
	2-09	32.4	3.90	1.21		5.80	1.79		0.96	0.30	
	2-10	18.0	2.57	1.43		3.48	1.93		0.83	0.46	
	2-11	7.2	0.60	0.84	1.30	1.03	1.42	1.81	0.19	0.27	0.39
					0.12			0.09			0.04
Co60-65D	3-06	32.5	4.60	1.41		6.69	2.05		1.12	0.34	
	3-07	19.4	2.49	1.28		4.08	2.11		0.77	0.40	
	3-08	16.0	2.02	1.26		3.39	2.12		0.63	0.39	
	3-09	18.3	3.14	1.72		4.08	2.23		0.96	0.52	
	3-10	23.9	3.11	1.30		4.77	1.99		0.76	0.32	
	3-10	7.7	0.88	1.15	1.35	1.06	1.37	1.98	0.50	0.65	0.44
					0.08			0.13			0.05
coldMn-65D	4-06	25.6	3.65	1.42		5.79	2.26		1.41	0.55	
	4-07	22.4	3.92	1.75		3.84	1.71		1.21	0.54	
	4-08	19.1	2.62	1.37		3.03	1.58		0.61	0.32	
	4-09	18.3	2.04	1.12		2.88	1.57		0.65	0.36	
	4-10	17.0	1.90	1.12		2.40	1.41		0.53	0.31	

	4-11	9.5	0.39	0.42	0.53	0.30	0.32	0.49	0.38	0.40	0.30
					0.04			0.06			0.03
C-65D	5-06	40.3	2.86	0.71		2.49	0.62		1.08	0.27	
	5-07	32.3	2.08	0.65		2.20	0.68		1.11	0.35	
	5-08	31.3	1.67	0.53		1.78	0.57		0.70	0.22	
	5-09	44.8	2.86	0.64		3.92	0.88		2.29	0.51	
	5-10	18.7	0.94	0.50		1.00	0.54		0.40	0.21	
	5-11	14.6	0.44	0.30	0.56	0.50	0.34	0.60	0.29	0.20	0.29
					0.06			0.07			0.05

	4-11	5.6	0.66	1.18	1.33	0.75	1.35	1.65	0.36	0.64	0.45
					0.10			0.13			0.06
C-65D	5-06	26.6	4.87	1.83		5.59	2.10		1.43	0.54	
	5-07	23.9	3.67	1.53		4.02	1.68		1.07	0.45	
	5-08	24.7	2.99	1.21		4.68	1.89		1.02	0.41	
	5-09	13.0	1.51	1.16		3.23	2.48		0.69	0.53	
	5-10	10.0	1.63	1.63		1.79	1.79		0.64	0.64	
	5-11	10.1	1.11	1.10	1.41	1.38	1.37	1.89	0.62	0.62	0.53
					0.12			0.15			0.04

	AQ1		AQ5		Smad7		AQ1		AQ5		Smad7	
Day 3	Mean	SE	Mean	SE	Mean	SE	Mean	SE	Mean	SE	Mean	SE
C-3D	1.00	0.057	0.65	0.075	0.53	0.088	1.95	0.226	1.97	0.284	0.73	0.050
Co60-3D	1.06	0.067	0.66	0.034	0.50	0.031	1.61	0.106	1.97	0.121	0.59	0.039
coldMn-3D	1.25	0.055	0.73	0.046	0.43	0.110	2.04	0.076	2.23	0.092	0.68	0.049
Mn56x1-3D	1.01	0.055	0.64	0.034	0.55	0.059	1.52	0.052	2.14	0.134	0.59	0.042
Mn56x3-3D	1.16	0.147	0.61	0.058	0.50	0.048	1.71	0.117	1.93	0.179	0.53	0.055

	Mean(%)	SE(%)	Mean(%)	SE(%)	Mean(%)	SE(%)	Mean(%)	SE(%)	Mean(%)	SE(%)	Mean(%)	SE(%)
C-3D	100	5.7	100	11.6	100	16.8	100	11.6	100	14.4	100	6.8
Co60-3D	106	6.7	103	5.2	95	5.8	83	5.5	100	6.1	81	5.3
coldMn-3D	125	5.5	113	7.1	81	20.8	104	3.9	113	4.7	92	6.7
Mn56x1-3D	102	5.5	99	5.3	104	11.3	78	2.7	109	6.8	81	5.8
Mn56x3-3D	116	14.8	94	8.9	95	9.1	87	6.0	98	9.1	72	7.5

Day 65												
C-65D	0.56	0.059	0.60	0.072	0.29	0.049	1.41	0.121	1.89	0.155	0.53	0.036
coldMn-65D	0.53	0.043	0.49	0.064	0.30	0.026	1.33	0.099	1.65	0.133	0.45	0.058
Co60-65D	0.51	0.079	0.54	0.076	0.34	0.030	1.35	0.080	1.98	0.125	0.44	0.051
Mn56x1-65D	0.61	0.026	0.65	0.036	0.35	0.019	1.18	0.098	1.81	0.108	0.49	0.042
Mn56x3-65D	0.78	0.086	0.58	0.082	0.35	0.043	1.30	0.115	1.81	0.086	0.39	0.040

	Mean(%)	SE(%)	Dunnett	Mean(%)	SE(%)	Mean(%)	SE(%)	Mean(%)	SE(%)	Mean(%)	SE(%)	Mean(%)	SE(%)
C-65D	100	10.6		100	12.0	100	16.5	100	8.6	100	8.2	100	6.9
Co60-65D	95	7.8		81	10.6	102	8.8	94	7.0	87	7.1	85	10.9
coldMn-65D	91	14.2		90	12.5	115	10.2	96	5.7	105	6.7	82	9.7
Mn56x1-65D	110	4.7	0.293	108	6.0	119	6.3	84	6.9	96	5.7	91	7.9
Mn56x3-65D	141	15.4	0.031	96	13.7	118	14.7	92	8.2	96	4.6	74	7.5
