# Supplementary material for: Effects of Radioactive 56MnO2 Particle Inhalation on Mouse Lungs: A Comparison between C57BL and BALB/c
Source: Int J Mol Sci. 2023 Dec 18;24(24):17605. doi: 10.3390/ijms242417605 (PMC10743477; doi:10.3390/ijms242417605)
Supplement: Supplementary file 1 [file ijms-24-17605-s001.zip › TableS1.pdf]

Body and Organ Weights - For Table 1

C57BL

Day3

	Stork #	B.W.	Organ weights Thymus	spleen	Lung	Heart	Liver	Kidney	Testis	Organ relative weights Thymus	spleen	Lung	Heart	Liver	Kidney	Testis
Mn56x1-3D	C1-1	25.9	0.04	0.14	0.22	0.16	1.55	0.35	0.16	1.5	5.4	8.5	6.2	59.8	13.5	6.2
	C1-2	27.7	0.03	0.12	0.25	0.16	1.44	0.41	0.21	1.1	4.3	9.0	5.8	52.0	14.8	7.6
	C1-3	28.3	0.04	0.14	0.29	0.18	1.68	0.45	0.16	1.4	4.9	10.2	6.4	59.4	15.9	5.7
	C1-4	31.7	0.04	0.14	0.29	0.17	1.51	0.44	0.21	1.3	4.4	9.1	5.4	47.6	13.9	6.6
	C1-5	33.9	0.04	0.15	0.27	0.19	1.91	0.54	0.11	1.2	4.4	8.0	5.6	56.3	15.9	3.2
	<M>	29.5								1.3	4.7	9.0	5.9	55.0	14.8	5.9
	<SE>	1.4								0.08	0.21	0.38	0.18	2.32	0.50	0.73
Mn56x3-3D	C2-1	26.4	0.03	0.07	0.3	0.12	1.39	0.37	0.24	1.1	2.7	11.4	4.5	52.7	14.0	9.1
	C2-2	28.2	0.04	0.1	0.25	0.13	1.48	0.38	0.14	1.4	3.5	8.9	4.6	52.5	13.5	5.0
	C2-3	28.5	0.05	0.13	0.25	0.13	1.66	0.4	0.2	1.8	4.6	8.8	4.6	58.2	14.0	7.0
	C2-4	30.5	0.04	0.1	0.32	0.16	1.63	0.55	0.21	1.3	3.3	10.5	5.2	53.4	18.0	6.9
	C2-5	33.4	0.05	0.08	0.29	0.16	1.98	0.49	0.15	1.5	2.4	8.7	4.8	59.3	14.7	4.5
	<M>	29.4								1.4	3.3	9.6	4.8	55.2	14.8	6.5
	<SE>	1.2								0.10	0.38	0.55	0.13	1.46	0.82	0.82
Co60-3D	C3-1	25.9	0.02	0.06	0.21	0.13	1.54	0.41	0.19	0.8	2.3	8.1	5.0	59.5	15.8	7.3
	C3-2	27.5	0.03	0.08	0.31	0.13	1.97	0.46	0.24	1.1	2.9	11.3	4.7	71.6	16.7	8.7
	C3-3	27.8	0.02	0.07	0.2	0.15	1.72	0.39	0.17	0.7	2.5	7.2	5.4	61.9	14.0	6.1
	C3-4	30.6	0.03	0.12	0.26	0.21	1.53	0.44	0.25	1.0	3.9	8.5	6.9	50.0	14.4	8.2
	C3-5	32.4	0.02	0.07	0.24	0.19	1.93	0.55	0.17	0.6	2.2	7.4	5.9	59.6	17.0	5.2
	<M>	28.8								0.8	2.8	8.5	5.6	60.5	15.6	7.1
	<SE>	1.2								0.09	0.32	0.73	0.37	3.45	0.60	0.64
coldMn-3D	C4-1	26.1	0.04	0.1	0.25	0.15	1.42	0.4	0.2	1.5	3.8	9.6	5.7	54.4	15.3	7.7
	C4-2	28.8	0.02	0.07	0.28	0.15	1.54	0.45	0.19	0.7	2.4	9.7	5.2	53.5	15.6	6.6
	C4-3	28.6	0.05	0.09	0.23	0.16	1.48	0.45	0.19	1.7	3.1	8.0	5.6	51.7	15.7	6.6
	C4-4	29.5	0.04	0.12	0.3	0.17	1.43	0.41	0.18	1.4	4.1	10.2	5.8	48.5	13.9	6.1
	C4-5	31.8	0.05	0.12	0.3	0.21	1.63	0.49	0.18	1.6	3.8	9.4	6.6	51.3	15.4	5.7
	<M>	29.0								1.4	3.5	9.4	5.8	51.9	15.2	6.5
	<SE>	0.9								0.18	0.30	0.36	0.23	1.02	0.33	0.33
C-3D	C5-1	25.9	0.04	0.08	0.19	0.15	1.51	0.41	0.19	1.5	3.1	7.3	5.8	58.3	15.8	7.3
	C5-2	27.6	0.03	0.07	0.26	0.16	1.55	0.38	0.19	1.1	2.5	9.4	5.8	56.2	13.8	6.9
	C5-3	28.5	0.04	0.09	0.25	0.16	1.7	0.41	0.17	1.4	3.2	8.8	5.6	59.6	14.4	6.0
	C5-4	31.4	0.06	0.06	0.4	0.19	1.25	0.53	0.23	1.9	1.9	12.7	6.1	39.8	16.9	7.3
	C5-5	30.2	0.08	0.11	0.39	0.16	1.46	0.49	0.19	2.6	3.6	12.9	5.3	48.3	16.2	6.3
	<M>	28.7								1.7	2.9	10.2	5.7	52.5	15.4	6.8
	<SE>	1.0								0.27	0.30	1.11	0.12	3.72	0.58	0.28

C57BL

Day65

		B.W.	Organ weights Thymus	spleen	Lung	Heart	Liver	Kidney	Testis	Organ relative weights Thymus	Lung	Heart	Liver	spleen	Kidney	Testis
Mn56x1-65D	C1-6	31.3	0.04	0.08	0.29	0.17	1.57	0.45	0.28	1.3	2.6	9.3	5.4	50.2	14.4	8.9
	C1-7	36.7	0.05	0.17	0.28	0.21	1.43	0.65	0.22	1.4	4.6	7.6	5.7	39.0	17.7	6.0
	C1-8	31.9	0.05	0.12	0.29	0.17	1.59	0.47	0.14	1.6	3.8	9.1	5.3	49.8	14.7	4.4
	C1-9	34.6	0.03	0.09	0.28	0.21	1.92	0.47	0.23	0.9	2.6	8.1	6.1	55.5	13.6	6.6
	C1-10	44.1	0.07	0.10	0.44	0.21	2.00	0.55	0.12	1.6	2.3	10.0	4.8	45.4	12.5	2.7
	C1-11	33.7	0.03	0.11	0.42	0.20	1.55	0.52	0.22	0.9	3.3	12.5	5.9	46.0	15.4	6.5
	<M>	35.4								1.3	3.2	9.4	5.5	47.6	14.7	5.9
	<SE>	2.10								0.14	0.40	0.77	0.21	2.50	0.80	0.95
Mn56x3-65D	C2-6	30.0	0.04	0.09	0.24	0.19	1.47	0.47	0.21	1.3	3.0	8.0	6.3	49.0	15.7	7.0
	C2-7	31.3	0.06	0.06	0.27	0.17	1.19	0.44	0.17	1.9	1.9	8.6	5.4	38.0	14.1	5.4
	C2-8	38.2	0.07	0.13	0.26	0.22	1.79	0.65	0.22	1.8	3.4	6.8	5.8	46.9	17.0	5.8
	C2-9	35.4	0.06	0.07	0.32	0.16	1.49	0.54	0.27	1.7	2.0	9.0	4.5	42.1	15.3	7.6
	C2-10	31.7	0.05	0.09	0.29	0.21	1.40	0.51	0.29	1.6	2.8	9.1	6.6	44.2	16.1	9.1
	C2-11	36.5	0.04	0.13	0.30	0.21	1.54	0.54	0.24	1.1	3.6	8.2	5.8	42.2	14.8	6.6
	<M>	33.9								1.6	2.8	8.3	5.7	43.7	15.5	6.9
	<SE>	1.47								0.14	0.31	0.38	0.33	1.74	0.46	0.61
Co60-65D	C3-6	30.5	0.04	0.11	0.24	0.17	1.55	0.48	0.18	1.3	3.6	7.9	5.6	50.8	15.7	5.9
	C3-7	32.3	0.04	0.07	0.26	0.16	1.58	0.44	0.23	1.2	2.2	8.0	5.0	48.9	13.6	7.1
	C3-8	32.5	0.03	0.10	0.32	0.18	1.79	0.51	0.23	0.9	3.1	9.8	5.5	55.1	15.7	7.1
	C3-9	33.2	0.05	0.14	0.30	0.24	1.28	0.56	0.21	1.5	4.2	9.0	7.2	38.6	16.9	6.3
	C3-10	32.8	0.05	0.13	0.23	0.20	1.55	0.58	0.21	1.5	4.0	7.0	6.1	47.3	17.7	6.4
	C3-11	34.2	0.05	0.12	0.27	0.22	1.67	0.57	0.24	1.5	3.5	7.9	6.4	48.8	16.7	7.0
	<M>	32.6								1.3	3.4	8.3	6.0	48.2	16.0	6.6
	<SE>	0.55								0.10	0.33	0.45	0.36	2.44	0.63	0.22
coldMn-65D	C4-6	32.1	0.05	0.11	0.19	0.19	1.74	0.41	0.28	1.6	3.4	5.9	5.9	54.2	12.8	8.7
	C4-7	29.9	0.05	0.12	0.23	0.18	1.65	0.45	0.15	1.7	4.0	7.7	6.0	55.2	15.1	5.0
	C4-8	33.8	0.04	0.09	0.31	0.21	1.37	0.64	0.20	1.2	2.7	9.2	6.2	40.5	18.9	5.9
	C4-9	34.0	0.05	0.09	0.23	0.19	1.73	0.53	0.18	1.5	2.6	6.8	5.6	50.9	15.6	5.3
	C4-10	35.8	0.04	0.11	0.27	0.22	1.69	0.66	0.22	1.1	3.1	7.5	6.1	47.2	18.4	6.1
	C4-11	35.9	0.06	0.08	0.23	0.17	1.70	0.64	0.22	1.7	2.2	6.4	4.7	47.4	17.8	6.1
	<M>	33.6								1.4	3.0	7.2	5.8	49.2	16.4	6.2
	<SE>	1.03								0.11	0.29	0.52	0.25	2.42	1.06	0.59
C-65D	C5-6	32.0	0.05	0.12	0.24	0.14	1.66	0.48	0.18	1.6	3.8	7.5	4.4	51.9	15.0	5.6
	C5-7	37.5	0.06	0.07	0.27	0.19	1.60	0.54	0.20	1.6	1.9	7.2	5.1	42.7	14.4	5.3
	C5-8	37.1	0.07	0.12	0.23	0.19	1.93	0.64	0.21	1.9	3.2	6.2	5.1	52.0	17.3	5.7
	C5-9	33.0	0.06	0.10	0.21	0.20	1.86	0.45	0.14	1.8	3.0	6.4	6.1	56.4	13.6	4.2
	C5-10	37.1	0.05	0.11	0.38	0.19	2.12	0.60	0.20	1.3	3.0	10.2	5.1	57.1	16.2	5.4
	C5-11	39.3	0.06	0.11	0.26	0.22	1.82	0.57	0.21	1.5	2.8	6.6	5.6	46.3	14.5	5.3
	<M>	36.0								1.6	2.9	7.4	5.2	51.1	15.2	5.3
	<SE>	1.27								0.09	0.28	0.67	0.25	2.53	0.59	0.23

BALB/c
Day3

		B.W.	Organ weights							Organ relative weights						
	Stork #		Thymus	spleen	Lung	Heart	Liver	Kidney	Testis	Thymus	spleen	Lung	Heart	Liver	Kidney	Testis
Mn56x1-3D	B1-1	22.2	0.02	0.1	0.24	0.14	1.12	0.39	0.21	0.9	4.5	10.8	6.3	50.5	17.6	9.5
	B1-2	23.5	0.03	0.09	0.2	0.13	1.17	0.4	0.23	1.3	3.8	8.5	5.5	49.8	17.0	9.8
	B1-3	26.1	0.04	0.12	0.22	0.14	1.3	0.45	0.23	1.5	4.6	8.4	5.4	49.8	17.2	8.8
	B1-4	29.7	0.05	0.1	0.24	0.19	1.56	0.43	0.28	1.7	3.4	8.1	6.4	52.5	14.5	9.4
	B1-5	33.5	0.05	0.14	0.28	0.19	1.95	0.49	0.17	1.5	4.2	8.4	5.7	58.2	14.6	5.1
	<M>	27.0								1.4	4.1	8.8	5.9	52.2	16.2	8.5
	<SE>	2.1								0.14	0.23	0.50	0.21	1.59	0.67	0.87
Mn56x3-3D	B2-1	21.9	0.02	0.12	0.19	0.12	1.24	0.38	0.18	0.9	5.5	8.7	5.5	56.6	17.4	8.2
	B2-2	25.8	0.02	0.08	0.21	0.14	1.36	0.44	0.12	0.8	3.1	8.1	5.4	52.7	17.1	4.7
	B2-3	25.9	0.03	0.12	0.25	0.13	1.45	0.43	0.26	1.2	4.6	9.7	5.0	56.0	16.6	10.0
	B2-4	29.5	0.03	0.1	0.23	0.17	1.6	0.49	0.21	1.0	3.4	7.8	5.8	54.2	16.6	7.1
	B2-5	33.2	0.03	0.19	0.35	0.18	2.15	0.64	0.24	0.9	5.7	10.5	5.4	64.8	19.3	7.2
	<M>	27.3								1.0	4.5	9.0	5.4	56.9	17.4	7.5
	<SE>	1.9								0.06	0.53	0.50	0.12	2.09	0.50	0.87
Co60-3D	B3-1	21.5	0.02	0.06	0.21	0.12	1.32	0.4	0.25	0.9	2.8	9.8	5.6	61.4	18.6	11.6
	B3-2	23.8	0.02	0.11	0.18	0.12	1.37	0.42	0.2	0.8	4.6	7.6	5.0	57.6	17.6	8.4
	B3-3	24.6	0.02	0.08	0.25	0.15	1.4	0.42	0.21	0.8	3.3	10.2	6.1	56.9	17.1	8.5
	B3-4	29.1	0.02	0.07	0.23	0.15	1.5	0.51	0.24	0.7	2.4	7.9	5.2	51.5	17.5	8.2
	B3-5	32.0	0.04	0.08	0.28	0.2	1.7	0.64	0.29	1.3	2.5	8.8	6.3	53.1	20.0	9.1
	<M>	26.2								0.9	3.1	8.8	5.6	56.1	18.2	9.2
	<SE>	1.9								0.09	0.40	0.51	0.24	1.74	0.52	0.63
coldMn-3D	B4-1	23.1	0.03	0.13	0.21	0.12	1.23	0.43	0.23	1.3	5.6	9.1	5.2	53.2	18.6	10.0
	B4-2	24.0	0.02	0.13	0.18	0.14	1.15	0.44	0.21	0.8	5.4	7.5	5.8	47.9	18.3	8.8
	B4-3	27.5	0.04	0.13	0.24	0.13	1.38	0.44	0.28	1.5	4.7	8.7	4.7	50.2	16.0	10.2
	B4-4	29.7	0.03	0.13	0.31	0.18	1.36	0.5	0.28	1.0	4.4	10.4	6.1	45.8	16.8	9.4
	B4-5	33.1	0.04	0.13	0.25	0.18	1.24	0.63	0.23	1.2	3.9	7.6	5.4	37.5	19.0	6.9
	<M>	27.5								1.2	4.8	8.7	5.5	46.9	17.8	9.1
	<SE>	1.8								0.11	0.32	0.54	0.24	2.67	0.58	0.58
C-3D	B5-1	22.3	0.03	0.1	0.21	0.12	1.01	0.35	0.21	1.3	4.5	9.4	5.4	45.3	15.7	9.4
	B5-2	23.5	0.03	0.08	0.18	0.13	1.09	0.37	0.25	1.3	3.4	7.7	5.5	46.4	15.7	10.6
	B5-3	26.6	0.03	0.09	0.22	0.14	1.21	0.52	0.21	1.1	3.4	8.3	5.3	45.5	19.5	7.9
	B5-4	28.2	0.04	0.08	0.33	0.18	1.44	0.48	0.25	1.4	2.8	11.7	6.4	51.1	17.0	8.9
	B5-5	30.9	0.06	0.15	0.27	0.17	1.8	0.53	0.22	1.9	4.9	8.7	5.5	58.3	17.2	7.1
	<M>	26.3								1.4	3.8	9.2	5.6	49.3	17.0	8.8
	<SE>	1.6								0.14	0.38	0.70	0.20	2.47	0.70	0.61

BALB/c
Day65

		B.W.	Organ weights							Organ relative weights						
			Thymus	spleen	Lung	Heart	Liver	Kidney	Testis	Thymus	Lung	Heart	Liver	spleen	Kidney	Testis
Mn56x1-65D	B1-6	24.2	0.03	0.12	0.25	0.15	1.31	0.49	0.19	1.2	5.0	10.3	6.2	54.1	20.2	7.9
	B1-7	24.3	0.03	0.14	0.28	0.17	1.48	0.52	0.06	1.2	5.8	11.5	7.0	60.9	21.4	2.5
	B1-8	29.5	0.04	0.08	0.28	0.16	1.19	0.48	0.28	1.4	2.7	9.5	5.4	40.3	16.3	9.5
	B1-9	30.3	0.03	0.09	0.29	0.18	1.51	0.53	0.23	1.0	3.0	9.6	5.9	49.8	17.5	7.6
	B1-10	28.3	0.04	0.11	0.27	0.14	1.31	0.5	0.22	1.4	3.9	9.5	4.9	46.3	17.7	7.8
	B1-11	33.5	0.03	0.11	0.25	0.19	1.47	0.58	0.25	0.9	3.3	7.5	5.7	43.9	17.3	7.5
	<M>	28.4								1.2	3.9	9.7	5.9	49.2	18.4	7.1
	<SE>	1.62								0.09	0.54	0.59	0.31	3.33	0.88	1.07
Mn56x3-65D	B2-6	23.5	0.03	0.1	0.13	0.23	1.06	0.47	0.28	1.3	4.3	5.5	9.8	45.1	20.0	11.9
	B2-7	25.0	0.03	0.08	0.23	0.13	1.14	0.45	0.23	1.2	3.2	9.2	5.2	45.6	18.0	9.2
	B2-8	26.7	0.03	0.14	0.27	0.18	1.24	0.56	0.31	1.1	5.2	10.1	6.7	46.4	21.0	11.6
	B2-9	26.3	0.04	0.1	0.26	0.16	1.3	0.52	0.2	1.5	3.8	9.9	6.1	49.4	19.8	7.6
	B2-10	30.0	0.03	0.1	0.3	0.17	1.44	0.49	0.21	1.0	3.3	10.0	5.7	48.0	16.3	7.0
	B2-11	33.2	0.03	0.13	0.33	0.2	1.78	0.64	0.24	0.9	3.9	9.9	6.0	53.6	19.3	7.2
	<M>	27.5								1.2	4.0	9.1	6.6	48.0	19.1	9.1
	<SE>	1.59								0.10	0.33	0.80	0.74	1.42	0.74	0.99
Co60-65D	B3-6	24.6	0.04	0.1	0.17	0.15	1.17	0.46	0.17	1.6	4.1	6.9	6.1	47.6	18.7	6.9
	B3-7	29.1	0.03	0.07	0.21	0.16	1.21	0.44	0.2	1.0	2.4	7.2	5.5	41.6	15.1	6.9
	B3-8	37.2	0.04	0.1	0.24	0.23	1.73	0.76	0.21	1.1	2.7	6.5	6.2	46.5	20.4	5.6
	B3-9	32.1	0.03	0.1	0.24	0.16	1.37	0.53	0.25	0.9	3.1	7.5	5.0	42.7	16.5	7.8
	B3-10	30.9	0.04	0.09	0.28	0.17	1.87	0.5	0.29	1.3	2.9	9.1	5.5	60.5	16.2	9.4
	B3-11	38.5	0.04	0.14	0.26	0.21	1.41	0.59	0.2	1.0	3.6	6.8	5.5	36.6	15.3	5.2
	<M>	32.1								1.2	3.1	7.3	5.6	45.9	17.0	7.0
	<SE>	2.31								0.11	0.28	0.42	0.20	3.64	0.94	0.68
coldMn-65D	B4-6	25.3	0.03	0.11	0.2	0.14	1.19	0.46	0.24	1.2	4.3	7.9	5.5	47.0	18.2	9.5
	B4-7	23.8	0.03	0.09	0.16	0.13	1.17	0.45	0.23	1.3	3.8	6.7	5.5	49.2	18.9	9.7
	B4-8	26.9	0.02	0.07	0.24	0.13	0.99	0.48	0.22	0.7	2.6	8.9	4.8	36.8	17.8	8.2
	B4-9	29.7	0.03	0.1	0.29	0.19	1.42	0.56	0.24	1.0	3.4	9.8	6.4	47.8	18.9	8.1
	B4-10	32.0	0.04	0.08	0.26	0.19	1.5	0.53	0.32	1.3	2.5	8.1	5.9	46.9	16.6	10.0
	B4-11	33.6	0.04	0.09	0.3	0.18	1.4	0.55	0.3	1.2	2.7	8.9	5.4	41.7	16.4	8.9
	<M>	28.6								1.1	3.2	8.4	5.6	44.9	17.8	9.1
	<SE>	1.73								0.09	0.33	0.47	0.24	2.11	0.49	0.36
C-65D	B5-6	22.8	0.03	0.08	0.29	0.13	0.97	0.44	0.23	1.3	3.5	12.7	5.7	42.5	19.3	10.1
	B5-7	24.6	0.02	0.09	0.22	0.28	1.12	0.43	0.22	0.8	3.7	8.9	11.4	45.5	17.5	8.9
	B5-8	34.2	0.05	0.14	0.28	0.23	1.66	0.57	0.22	1.5	4.1	8.2	6.7	48.5	16.7	6.4
	B5-9	30.2	0.05	0.12	0.24	0.17	1.73	0.59	0.3	1.7	4.0	7.9	5.6	57.3	19.5	9.9
	B5-10	30.0	0.02	0.11	0.24	0.17	1.39	0.5	0.22	0.7	3.7	8.0	5.7	46.3	16.7	7.3
	B5-11	37.2	0.04	0.1	0.29	0.21	1.99	0.69	0.36	1.1	2.7	7.8	5.6	53.5	18.5	9.7
	<M>	29.8								1.2	3.6	8.9	6.8	49.0	18.0	8.7
	<SE>	2.45								0.17	0.22	0.85	1.02	2.45	0.57	0.68
