# Supplementary material for: Effects of Radioactive 56MnO2 Particle Inhalation on Mouse Lungs: A Comparison between C57BL and BALB/c
Source: Int J Mol Sci. 2023 Dec 18;24(24):17605. doi: 10.3390/ijms242417605 (PMC10743477; doi:10.3390/ijms242417605)
Supplement: Supplementary file 1 [file ijms-24-17605-s001.zip › TableS2.pdf]

Q-PCR results for Figure 2

C57BL-Lung

**Day 3**			Bax			Phlda3			Fam3		
		Average		x10/bact			x10/bact			x10/bact	
Mn56x1-3I	C1-01	26.0	1.07	0.41		2.84	1.10		1.08	0.42	
	1-02	21.4	0.95	0.44		1.62	0.76		0.56	0.26	
	1-03	21.9	1.12	0.51		2.12	0.97		0.56	0.26	
	1-04	13.0	0.58	0.44		1.00	0.77		0.58	0.45	
	1-05	15.2	0.92	0.61	0.48	1.52	1.00	0.92	0.47	0.31	0.34
					0.04			0.07			0.04
Mn56x3-3I	2-01	16.1	1.08	0.67		1.92	1.20		0.22	0.14	
	2-02	18.8	1.01	0.54		1.48	0.79		0.46	0.25	
	2-03	26.8	1.54	0.58		3.50	1.31		2.19	0.82	
	2-04	20.0	1.11	0.55		2.21	1.10		0.35	0.18	
	2-05	10.6	0.44	0.42	0.55	0.70	0.66	1.01	0.23	0.22	0.32
					0.04			0.12			0.13
Co60-3D	3-01	27.8	3.08	1.11		6.75	2.43		0.36	0.13	
	3-02	20.6	1.73	0.84		2.99	1.45		0.18	0.09	
	3-03	15.2	1.52	1.00		2.99	1.96		0.14	0.09	
	3-04	11.8	0.82	0.70		2.07	1.75		0.19	0.16	
	3-05	15.1	1.40	0.93	0.91	2.51	1.67	1.85	0.16	0.11	0.12
					0.07			0.17			0.01
coldMn-3I	4-01	20.9	1.27	0.61		2.58	1.23		0.75	0.36	
	4-02	17.4	1.04	0.60		1.31	0.75		0.33	0.19	
	4-03	23.5	1.11	0.47		1.87	0.80		1.11	0.47	
	4-04	30.0	1.40	0.47		3.10	1.03		1.13	0.38	
	4-05	9.8	0.47	0.48	0.53	1.09	1.12	0.99	0.47	0.48	0.38
					0.03			0.09			0.05
C-3D	5-01	5.3	0.26	0.49		0.29	0.55		0.25	0.48	
	5-02	14.0	0.68	0.48		0.86	0.61		0.56	0.40	
	5-03	13.7	0.74	0.54		0.92	0.67		0.34	0.25	
	5-04	11.3	0.49	0.43		0.75	0.67		0.55	0.49	
	5-05	8.7	0.33	0.38	0.47	1.27	1.46	0.79	0.55	0.63	0.45
					0.03		1.11	0.17		0.32	0.06

	Mean	SE	Mean	SE	Mean	SE
C-3D	0.47	0.027	0.79	0.169	0.45	0.063
Co60-3D	0.91	0.070	1.85	0.166	0.12	0.014
coldMn-3D	0.53	0.032	0.99	0.092	0.38	0.053
Mn56x1-3D	0.48	0.035	0.92	0.067	0.34	0.040
Mn56x3-3D	0.55	0.040	1.01	0.123	0.32	0.126

	Mean(%)	SE(%)	Dunnett	Mean(%)	SE(%)	Dunnett	Mean(%)	SE(%)	Dunnett
C-3D	100	5.9		100	21.3		100	14.0	
Co60-3D	196	15.0	0.003	233	20.9	0.004	26	3.2	0.008
coldMn-3D	113	6.9		124	11.5		84	11.7	
Mn56x1-3D	104	7.6		116	8.4		76	9.0	
Mn56x3-3D	118	8.7		128	15.5		71	28.2	

BalbC-Lung

**Day 3**		Bact	bax			Phlda3			Faim3		
		Average	11/4-23	x10/bact			x10/bact			x10/bact	
Mn56x1-3I	B1-01	42.4	1.05	0.25		1.03	0.24		1.44	0.34	
	1-02	34.3	1.07	0.31		1.15	0.34		1.96	0.57	
	1-03	29.8	0.98	0.33		0.84	0.28		2.15	0.72	
	1-04	29.2	0.94	0.32		1.11	0.38		2.37	0.81	
	1-05	19.1	0.88	0.46	0.34	0.41	0.21	0.29	1.71	0.89	0.67
					0.03			0.03			0.10
Mn56x3-3I	2-01	17.6	0.51	0.29		0.47	0.27		1.45	0.83	
	2-02	20.9	0.61	0.29		0.60	0.29		3.28	1.57	
	2-03	22.9	0.98	0.43		1.11	0.49		2.34	1.02	
	2-04	29.1	0.73	0.25		1.01	0.35		1.35	0.47	
	2-05	34.1	0.87	0.26	0.30	0.63	0.19	0.31	1.02	0.30	0.84
					0.03			0.05			0.22
Co60-3D	3-01	30.5	1.61	0.53		1.40	0.46		1.11	0.36	
	3-02	13.4	0.73	0.54		0.44	0.33		0.35	0.26	
	3-03	21.8	1.08	0.50		0.92	0.42		0.84	0.39	
	3-04	17.8	0.92	0.51		0.77	0.43		1.01	0.57	
	3-05	15.0	0.73	0.48	0.51	0.60	0.40	0.41	1.41	0.94	0.50
					0.01			0.02			0.12
coldMn-3I	4-01	33.0	1.01	0.31		0.85	0.26		4.91	1.49	
	4-02	25.4	0.70	0.28		0.69	0.27		2.79	1.10	
	4-03	12.5	0.32	0.26		0.28	0.23		2.65	2.11	
	4-04	24.6	0.59	0.24		0.56	0.23		5.88	2.39	
	4-05	21.0	0.52	0.25	0.27	0.48	0.23	0.24	3.34	1.59	1.74
					0.01			0.01			0.23
C-3D	5-01	15.8	0.53	0.34		0.38	0.24		2.93	1.85	
	5-02	3.1	0.12	0.39		0.05	0.15		0.54	1.73	
	5-03	10.3	0.21	0.20		0.21	0.20		2.19	2.12	
	5-04	17.1	0.55	0.32		0.36	0.21		2.85	1.66	
	5-05	6.5	0.17	0.26	0.30	0.15	0.23	0.21	0.73	1.13	1.70
					0.03			0.02			0.16

	Mean	SE	Dunnett	Mean	SE	Dunnett	Mean	SE	Dunnett
C-3D	0.30	0.032		0.21	0.017		1.70	0.162	
Co60-3D	0.51	0.011		0.41	0.022		0.50	0.119	
coldMn-3D	0.27	0.012		0.24	0.009		1.74	0.231	
Mn56x1-3D	0.34	0.035		0.29	0.031		0.67	0.098	
Mn56x3-3D	0.30	0.032		0.31	0.050		0.84	0.224	

	Mean(%)	SE(%)	Dunnett	Mean(%)	SE(%)	Dunnett	Mean(%)	SE(%)	Dunnett
C-3D	100	10.5		100	8.1		100	9.6	
Co60-3D	171	3.5	0.005	199	10.5	0.000	30	7.0	
coldMn-3D	88	3.9		118	4.5		102	13.6	0.001
Mn56x1-3D	111	11.6		141	15.0		39	5.8	0.009
Mn56x3-3D	101	10.7		153	24.4		49	13.2	0.033
